# Oxidized dietary lipids induce vascular inflammation and atherogenesis in post-menopausal rats: estradiol and selected antihyperlipidemic drugs restore vascular health in vivo

**DOI:** 10.1186/s12944-023-01818-y

**Published:** 2023-07-26

**Authors:** Joy Temiloluwa Folahan, Olufunke Esan Olorundare, Abayomi Mayowa Ajayi, Adeoye Oyetunji Oyewopo, Sunday Sokunle Soyemi, Adejuwon Adewale Adeneye, Ikechukwu Innocent Okoye, Saheed Olanrewaju Afolabi, Anoka Ayembe Njan

**Affiliations:** 1 School of Basic Pharmaceutical and Toxicological Sciences, College of Pharmacy, University of Louisiana Monroe, Monroe, LA 71209 USA; 2 Department of Pharmacology and Therapeutics, Faculty of Basic Clinical Sciences, University of Ilorin, Ilorin, Kwara-State Nigeria; 3 Department of Pharmacology and Therapeutics, Faculty of Basic Medical Sciences, University of Ibadan, Ibadan, Oyo-State Nigeria; 4 Department of Anatomy, Faculty of Basic Medical Sciences, University of Ilorin, Ilorin, Kwara- State Nigeria; 5 Department of Pathology and Forensic Medicine, Faculty of Basic Clinical Sciences, Lagos State University College of Medicine, Ikeja, Nigeria; 6 Department of Pharmacology, Therapeutics and Toxicology, Faculty of Basic Clinical Sciences, Lagos State University College of Medicine, Ikeja, Nigeria; 7 Department of Oral Pathology and Medicine, Faculty of Dentistry, Lagos State University College of Medicine, Ikeja, Nigeria

**Keywords:** Oxidized lipids, Vascular inflammation, Atherogenesis, Estrogen, Menopause, Antihyperlipidemic drugs

## Abstract

**Background:**

Thermoxidation of edible oil through deep fat frying results in the generation of several oxidized products that promote lipid peroxidation and ROS production when eaten. Consumption of thermoxidized oil in post-menopausal conditions where the estrogen level is low contributes to cardiovascular disease. This study evaluates the role of estradiol and antihyperlipidemic agents (AHD) in restoring the vascular health of ovariectomized (OVX) rats fed with thermoxidized palm oil (TPO) and thermoxidized soya oil (TSO) diets.

**Method:**

A total of 10 groups of rats (n = 6) were set up for the experiment. Group I (normal control) rats were sham handled while other groups were OVX to bring about estrogen deficient post-menopausal state. Group II (OVX only) was not treated and received normal rat chow. Groups III-X were fed with either TPO or TSO diet for 12 weeks and treated with estradiol (ETD) 0.2 mg/kg/day, atorvastatin (ATV) 10 mg/kg/day, and a fixed-dose combination of ezetimibe and ATV (EZE 3 mg/kg/day + ATV 10 mg/kg/day).

**Results:**

Pro-atherogenic lipids levels were significantly elevated in untreated TSO and TPO groups compared to OVX and sham, resulting in increased atherogenic and Coronary-risk indices. Treatment with Estradiol and AHDs significantly reduced the total cholesterol, triglycerides, low-density lipoprotein cholesterol as well as AI and CRI compared to untreated TSO and TPO groups, whereas TSO and TPO groups showed significant elevation in these parameters compared to Group I values. Moreover, aortic TNF-α levels were extremely elevated in the untreated TSO and TPO compared to Group I. TNF-α levels were significantly reduced in rats treated with AHDs and ETD. Localized oxidative stress was indicated in the aortic tissues of TSO and TPO-fed OVX rats by increased malondialdehyde and decreased glutathione, catalase, and superoxide dismutase levels. This contributed to a depletion in aortic nitric oxide. AHDs and ETD replenished the nitric oxide levels significantly. Histological evaluation of the aorta of TSO and TPO rats revealed increased peri-adventitia fat, aortic medial hypertrophy, and aortic recanalization. These pathologic changes were less seen in AHDs and ETD rats.

**Conclusion:**

This study suggests that ETD and AHDs profoundly attenuate oxidized lipid-induced vascular inflammation and atherogenesis through oxidative-stress reduction and inhibition of TNF-α signaling.

## Background

Intermittent deep frying of edible oils is a common food processing practice in homes, restaurants, food stalls, and industries adopted to reduce cost and improve taste [1, 2]. In deep-fat frying, the oil is usually heated to an extreme temperature of 180 °C and higher in the presence of moisture and air, resulting in an array of chemical reactions known as lipid oxidation [3]. Thermal oxidation of dietary oils destroys essential fatty acids and generates products such as peroxides, hydroperoxides, aldehydes, and several other potentially hazardous non-volatile polar compounds that are retained in the thermoxidised oil and when consumed repeatedly increase the risk for cardiovascular diseases [1, 4, 5].

Atherosclerosis is an inflammatory disorder of large and medium-sized arteries. Endothelial dysfunction, vascular inflammation, and lipid deposition in the tunica intima are some of the features of atherosclerotic development and progression [6]. Oxidized-low density lipoprotein (Ox-LDL), a product of LDL-c oxidation, is associated with several pathological manifestations including atherosclerosis [5, 7]. In the early phase of atherosclerosis, LDL-c is deposited in the intima of the arterial wall and recruits inflammatory cells to the sub-endothelium. It is also involved in the formation of Ox-LDL, whose interaction with inflammatory cells results in the generation of reactive oxygen species (ROS) and depletion of Nitric oxide (NO) [7, 8]. Endothelial dysfunction and impaired NO release disrupt the regulation of vascular tone, creating an imbalance between vasoconstriction and vasodilation, resulting in atherogenesis [7, 9]. Structural changes observed in atherosclerotic arterial tissues include accumulation of blood cells in the luminal aspect of the aortic wall, leukocyte infiltration, aortic medial hypertrophy, and intimal plaques [6, 10].

There is a noticeable increase in risk for cardiovascular diseases (CVDs) including atherosclerosis in postmenopausal women when estrogen levels decline. While CVD is not prevalent in young women, it is the leading cause of morbidity and mortality in women above 50 years and accounts for about 75% of deaths in postmenopausal women [11]. Some mechanisms have been proposed to delineate the cardioprotective role of estrogen in premenopausal women, namely, estrogen administration positively impacts plasma lipid profiles, anti-platelet, and antioxidant status [12]. A Previous study revealed greater arterial stiffening, a known indicator of vascular aging in postmenopausal women when compared with pre-menopausal women alongside estrogen-linked endothelial-dependent vasodilatation [13]. The role of estradiol in the inhibition of monocyte adhesion to vascular endothelium, which is a critical step in the development of atheromas and arteriosclerosis has been established [14]. A synergy of these mechanisms which confers cardioprotective prowess to premenopausal women in the presence of estrogen invariably leads to a myriad of cardiac pathologies in postmenopausal women with very low levels of estrogen.

Women are around 10 years older than men at the first presentation of atherosclerotic cardiovascular disease, and this is related to the detrimental effect of estrogen withdrawal on cardiovascular functions and metabolism [15–17]. Several risk factors of cardiovascular disease are altered following the menopausal transition, resulting in a significant increase in the risk for myocardial infarction and cerebrovascular disease. These factors include dyslipidemia, hypertension, inflammation, and hemostatic factors [18, 19]. While post-menopausal status decreases vascular protection due to reduced estrogen levels, the consumption of thermoxidized oil further diminishes vascular integrity via oxidative stress and increased levels of oxidized lipids [3, 4].

Dyslipidemia is prominent among the surrogate markers of increased cardiovascular risks in postmenopausal women [16]. Despite the availability of various lipid-lowering drugs, reduction in cardiovascular risk and prevention of atherosclerosis in postmenopausal females has proven to be a tough challenge [2, 16]. Epidemiological studies clearly show that cardiovascular disease is the major cause of mortality in women in developed countries accounting for deaths of 75% or greater in postmenopausal women [11, 16].

This study examines estradiol and selected antihyperlipidemic drugs used singly or in combination for their athero-protective potentials in ovariectomized rats fed with thermoxidized oil diet.

## Methods

### Preparation of thermoxidised oil diet

Five liters of palm oil (Ace Products and Services Ltd., Nigeria) and soya oil (Sunola Foods Ltd., Plot 122–132, Oshodi-Apapa Expressway, Lagos State, Nigeria) were processed by repeated heating on a hot plate at 180°C for 20 min [20]. One and a half liters of oil was used to fry 2 kg of sweet potatoes in five cycles. At the end of each frying batch, the oil was cooled to room temperature (25° C) for 5 h and used to fry a fresh batch of potatoes for the second time without replacing lost oils. The cycle was repeated five times to make five times heated oil for the thermoxidized oil diet. Thermoxidized palm/soya oil (15% w/w) was formulated into diets with standard rat chow (Ladokun Feeds, Ibadan, Oyo State, Nigeria).

### Experimental animals

Healthy female Wistar rats (200–250 g) obtained from a private breeder (McTemmy Farms, Ogbomosho, Oyo State, Nigeria) were used for this study. The animals were housed individually in plastic cages at room temperature of 27 ± 2°C at the University of Ilorin, Central Laboratory Animal House under a 12-hour light/dark cycle and had *ad libitum* access to food and water. Institutional ethical approval for this study was obtained from the Ethical Review Committee of the University of Ilorin, with approval number UERC/ASN/2021/2038.

### Ovariectomy procedure and treatment

Ovariectomy was performed under ketamine (43.5 mg/kg) /xylazine (6.5 mg/kg) anesthesia injected intraperitoneally [21]. Skin and muscle layers were breached with incisions to gain access to the abdominal cavity, after which the fat bed embedding the ovary was identified and externalized. To initiate an estrogen-deficient postmenopausal state, the ovary was excised while the uterus and the abdominal fat bed were placed back into the cavity. Incisions were closed with a 4/0 absorbable suture. These steps were repeated to remove the contralateral ovary. Penicillin ointment was applied to the incision site to minimize infection while ofloxacin 1000 mg/kg was administered orally to the rats for seven days [22]. The rats were allowed free access to food and water for two weeks to recover from surgery and attain a post-menopausal state before drug treatments and formulated thermoxidized oil diets were instituted.

The experimental animals were randomly divided into ten groups (n = 6). Group I was sham-handled to simulate surgical stress, group II was ovariectomized and both groups were fed with standard rat chow while the other eight groups (Groups III-X) were ovariectomized (OVX) and fed with either thermoxidized palm oil (TPO) or thermoxidized Soya oil (TSO) diets. Estradiol and antihyperlipidemic drugs (AHDs) were orally administered as follows: estradiol valerate (ETD) 0.2 mg/kg/day, atorvastatin (ATV) 10 mg/kg/day and fixed-dose combination of ezetimibe and ATV (EZE 3 mg/kg/day + ATV 10 mg/kg/day). The animals were treated for 12 weeks at the end of which aortic tissues were harvested for biochemical and histological analysis.

### Sacrifice of experimental animals

At the end of treatment for a period of 12 weeks, the rats were fasted for 12 h and anesthetized with diethyl ether, and blood was obtained through cardiac puncture into heparinized tubes. The aortas were identified and harvested, rinsed in Tris-KCl buffer (0.15 M, pH 7.4) and frozen. Whole blood in heparin tubes was left standing at room temperature for 30 min and centrifuged at 800 g for 15 min to obtain plasma. The tissues were homogenized using a mechanical homogenizer with Teflon at 4 °C in sodium phosphate buffer (0.1 M, pH 7.4). The tissue homogenates were centrifuged at 2000 g for 10 min at 4 °C. The supernatants were aliquoted and stored at -20 °C for biochemical assays.

### Plasma lipid profile analyses

Total cholesterol (TC), Triglycerides (TG) and high-density lipoprotein-cholesterol (HDL-c) were assayed in plasma using Randox kits (Randox Laboratories Limited, United Kingdom). Very low-density lipoprotein cholesterol (VLDL-c) and low-density lipoprotein cholesterol (LDL-c) levels [23], atherogenic index (AI) [24], coronary risk index (CRI) [25] and HDL-c/LDL-c ratio [23] were calculated from the lipid profile.

### Determination of aortic nitric oxide and tumor necrosis factor-alpha (TNF-α)

Nitrite was measured as an indicator of nitric oxide (NO) production using the Griess method as previously reported [26]. Aortic TNF- α level was determined using the BioLegend ELISA kit (BioLegend Biotechnology Research, San Diego, California, U.S.A.) according to the manufacturer’s instructions.

### Aortic tissue oxidative stress assessment

Aortic tissue homogenates supernatants were used for oxidative stress assessment. Malondialdehyde (MDA) was measured as an index of lipid peroxidation using the assay of thiobarbituric reacting substances (TBARs) [27]. Reduced glutathione (GSH) as a non-enzymic antioxidant maker was measured using the method reported in [28]. Catalase (CAT) activity in the supernatants was determined using the colorimetric assay based on the yellow complex with molybdate and H_2_O_2_ as described by [29]. Superoxide dismutase (SOD) activity was determined using the method previously reported by Misra and Fridovich [30].

### Histological analysis

Ascending and thoracic aorta tissues were dehydrated and embedded in paraffin wax. Thin sections (5 μm) of the aortic tissues were cut and stained with hematoxylin and eosin (H &E) stain for light microscopy and viewed at x100 magnification.

### Statistical analysis

All data were presented as mean ± standard error mean (SEM). Normally distributed data were analyzed by parametric test using One-way analysis of variance (ANOVA) and Bonferroni’s multiple comparisons test. The level of significance was set at *p* < 0.05. Statistical analysis was performed by using GraphPad Prism 8 software.

## Results

### Effect of selected antihyperlipidemic drugs and estradiol on plasma lipid profile and atherogenic and coronary artery risk indices of ovariectomized rats fed with TPO and TSO

TC, TG, and LDL-c were significantly increased in untreated OVX + TSO (Table 1) and OVX + TPO rats (Table 2) compared to sham. Treatment with ETD and ATV significantly (*p* < 0.05) reduced these parameters compared to untreated OVX + TSO (Table 1). In OXV + TPO rats (Table 2), ETD, ATV and EZE + ATV treatments decreased TC and TG levels significantly (*p* < 0.05), and LDL-c levels were decreased by ATV treatment.

**Table 1:** Effects of estradiol and selected antihyperlipidemic drugs on plasma lipid profile of ovariectomized Wistar rats fed with thermoxidized soya oil diet

Treatment	Plasma Lipid profile parameters (mg/dL)				
	TC	TG	HDL-c	LDL-c	VLDL-c
Sham (Group I)	77.72 ± 1.29	96.23 ± 10.22	22.08 ± 0.77	36.40 ± 2.24	19.25 ± 2.04
OVX	93.58 ± 4.76	104.12 ± 2.01	21.18 ± 0.79	49.62 ± 0.86	21.3 ± 1.34
OVX + TSO	107.89 ± 1.68^**#**^	139.72 ± 21.97^**#**^	19.88 ± 1.21	60.07 ± 7.01^#^	27.94 ± 4.39^#^
OVX + TSO + ATV	82.35 ± 2.07***	103.20 ± 10.59***	24.53 ± 4.08**	37.17 ± 7.69***	20.64 ± 2.12*
OVX + TSO + EZE + ATV	88.68 ± 2.13***	97.22 ± 18.33***	20.67 ± 1.61	57.21 ± 10.10	19.44 ± 3.67**
OVX + TSO + ETD	76.05 ± 0.94***	104.26 ± 14.06***	20.88 ± 1.64	34.31 ± 10.02***	20.85 ± 2.81*

Values are expressed as mean ± SEM (n = 6)

#*p* < 0.05 compared to Group I values accepted as significant

*p < 0.05 compared to OVX + TPO

**p < 0.01 compared to OVX + TPO

***p < 0.001 compared to OVX + TPO

*OVX – ovariectomized; TSO – thermoxidized soya oil; ATV – atorvastatin; EZE – ezetimibe; ETD – estradiol; TC – total cholesterol; TG – triglycerides; HDL-c – high density lipoprotein cholesterol; LDL-c – low density lipoprotein cholesterol; VLDL-c – very low density lipoprotein cholesterol*

**Table 2:** Effects of estradiol and selected antihyperlipidemic drugs on plasma lipid profile of ovariectomized Wistar rats fed with thermoxidized palm oil diet

Treatment	Plasma Lipid profile parameters (mg/dL)				
	T-CHOL	TRIG	HDL	LDL	VLDL
Sham (Group I)	77.72 ± 1.29	96.23 ± 10.22	22.08 ± 0.77	36.40 ± 2.24	19.25 ± 2.04
OVX	93.58 ± 4.76	104.12 ± 2.01	21.18 ± 0.79	49.62 ± 0.86	21.3 ± 1.34
OVX + TPO	106.95 ± 2.70^**#**^	163.95 ± 16.53^**#**^	20.92 ± 0.60	55.66 ± 4.73^**#**^	30.37 ± 3.63^**#**^
OVX + TPO + ATV	82.49 ± 2.25***	110.45 ± 18.34***	21.48 ± 1.54	38.92 ± 4.62***	22.09 ± 3.67**
OVX + TPO + EZE + ATV	98.54 ± 0.66	114.89 ± 6.68***	20.73 ± 0.69	54.83 ± 1.95	22.98 ± 1.34*
OVX + TPO + ETD	95.33 ± 1.69**	126.19 ± 12.78*	19.54 ± 1.25	50.55 ± 5.32	25.24 ± 2.56

Values are expressed as mean ± standard error of mean (n = 6)

#*p* < 0.05 compared to Sham accepted as significant

*p < 0.05 compared to OVX + TPO

**p < 0.01 compared to OVX + TPO

***p < 0.001 compared to OVX + TPO

*OVX – ovariectomized; TPO – thermoxidized palm oil; ATV – atorvastatin; EZE – ezetimibe; ETD – estradiol; TC – total cholesterol; TG – Triglycerides; HDL-c – high density lipoprotein cholesterol; LDL-c – low density lipoprotein cholesterol; VLDL-c – very low density lipoprotein cholesterol*.

**Table 3:** Effects of estradiol and selected antihyperlipidemic drugs on plasma atherogenic indices of ovariectomized Wistar rats fed with thermoxidized soya oil diet

Treatment	Plasma Atherogenic indices		
	AI	CRI	HDL-c/LDL-c
Sham (Group I)	3.44 ± 0.59	3.55 ± 0.22	0.62 ± 0.05
OVX	4.71 ± 0.55	4.65 ± 0.33^**#**^	0.3 ± 0.04
OVX + TSO	6.03 ± 1.02^**#**^	5.57 ± 0.49^**#**^	0.36 ± 0.06
OVX + TSO + ATV	3.43 ± 0.41***	3.74 ± 0.52***	1.25 ± 0.32***
OVX + TSO + EZE + ATV	3.86 ± 0.94***	4.90 ± 0.67	0.45 ± 0.11
OVX + TSO + ETD	4.04 ± 0.64**	3.64 ± 0.38***	0.18 ± 0.31

Values are expressed as mean ± SEM (n = 6)

#*p* < 0.05 compared to Group I values accepted as significant

*p < 0.05 compared to OVX + TSO

**p < 0.01 compared to OVX + TSO

***p < 0.001 compared to OVX + TSO

*OVX – ovariectomized; TSO – thermoxidized soya oil; ATV – atorvastatin; EZE – ezetimibe; ETD – estradiol; HDL-c – high density lipoprotein cholesterol; LDL-c – low density lipoprotein cholesterol; AI – atherogenic index; CRI – coronary risk index*

**Table 4:** Effects of estradiol and selected antihyperlipidemic drugs on plasma atherogenic indices of ovariectomized Wistar rats fed with thermoxidized palm oil diet

Treatment	Plasma Atherogenic indices		
	AI	CRI	HDL-c/LDL-c
Sham (Group I)	3.44 ± 0.59	3.55 ± 0.22	0.62 ± 0.05
OVX	4.71 ± 0.55	4.65 ± 0.33^**#**^	0.3 ± 0.04
OVX + TPO	6.29 ± 0.92^**#**^	5.09 ± 0.21^**#**^	0.38 ± 0.02
OVX + TPO + ATV	4.21 ± 0.81***	3.88 ± 0.20**	0.57 ± 0.06
OVX + TPO + EZE + ATV	4.58 ± 0.39*	4.78 ± 0.17	0.38 ± 0.02
OVX + TPO + ETD	4.39 ± 0.29**	5.03 ± 0.52	0.42 ± 0.06

Values are expressed as mean ± standard error of mean (n = 6)

# - p < 0.05 compared to Sham accepted as significant

* - p < 0.05 compared to OVX + TPO

** - p < 0.01 compared to OVX + TPO

*** - p < 0.001 compared to OVX + TPO

*OVX – ovariectomized; TPO – thermoxidized palm oil; ATV – atorvastatin; EZE – ezetimibe; ETD – estradiol; HDL-c – high density lipoprotein cholesterol; LDL-c – low density lipoprotein cholesterol; AI – atherogenic index; CRI – coronary risk index.*

AI and CRI levels were significantly (*p* < 0.05) increased in OVX + TSO (Table 3) and OVX + TPO (Table 4) rats when compared with sham. ETD, ATV and EZE + ATV treatments lowered AI significantly (*p* < 0.05) in OVX + TSO and OVX + TPO diet groups. Similarly, CRI level was significantly (*p* < 0.05) decreased by ATV treatment in both diet groups when compared with sham. ETD treatment equally decreased the CRI, albeit in OVX + TSO diet group only (Table 3).

### Effect of selected antihyperlipidemic drugs and estradiol on aortic nitrites of ovariectomized rats fed with TPO and TSO

**Fig. 1:**
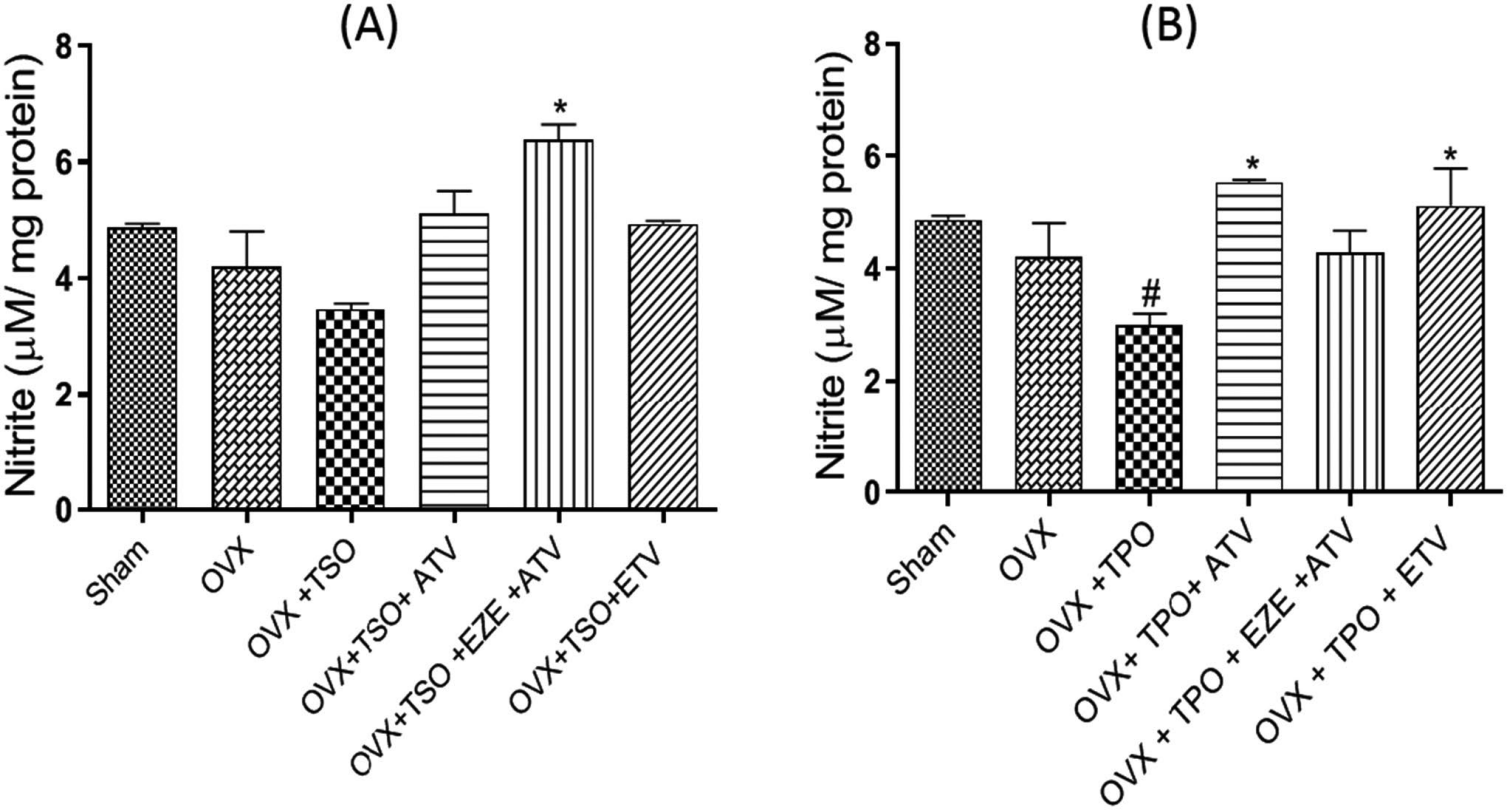
**Effects of estradiol and selected antihyperlipidemic drugs on aortic nitric oxide levels in ovariectomized Wistar rats fed with thermoxidized soya oil diet (A) and thermoxidized palm oil diet (B)** Values are expressed as mean ± standard error of mean (n = 3) #*p* < 0.05 compared to Sham accepted as significant *p < 0.05 compared to OVX + TPO/OVX + TSO *OVX – ovariectomized; TPO – thermoxidized palm oil; TSO – thermoxidized soya oil; ATV – atorvastatin; EZE – ezetimibe; ETD – estradiol*

Aorta tissue nitrites level was measured as an index for nitric oxide (NO) as shown in Fig. 1a and lb. There was a significant reduction in aortic nitrites levels in OVX + TPO groups when compared with Group I rats. Treatment with ATV and ETD significantly increased aortic nitrite levels when compared with OVX + TPO (Fig. 1b). In OVX + TSO diet groups, nitrite level was significantly increased in the EZE + ATV treated group (Fig. 1a).

### Effect of selected antihyperlipidemic drugs and estradiol on aortic TNF-α levels of ovariectomized rats fed with TPO and TSO

**Fig. 2:**
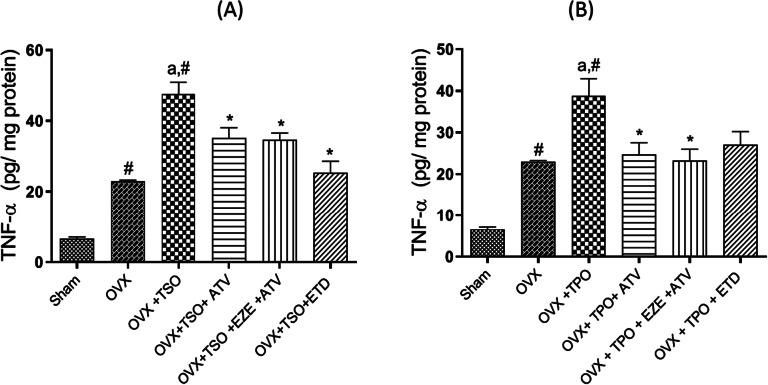
**Effects of estradiol and selected antihyperlipidemic drugs on aortic TNF-α levels in ovariectomized Wistar rats fed with thermoxidized soya oil diet (A) and thermoxidized palm oil diet (B)** Values are expressed as mean ± standard error of mean (n = 3) #*p* < 0.05 compared to Sham accepted as significant ^a^*p* < 0.05 compared to OVX *p < 0.05 compared to OVX + TPO/OVX + TSO *OVX – ovariectomized; TPO – thermoxidized palm oil; TSO – thermoxidized soya oil; ATV – atorvastatin; EZE – ezetimibe; ETD – estradiol; TNF-α – tumor necrosis factor-alpha*

As shown in Fig. 2a, TNF-α levels were significantly (*p* < 0.05) increased in OVX + TSO rats by about 80% compared to sham. However, treatment with ATV, EZE + ATV and ETD significantly (*p* < 0.05) decreased the TNF-α levels. Similarly, as shown in Fig. 2b, TNF-α levels were significantly (*p* < 0.05) elevated in OVX + TPO rats compared to sham. Treatment with ATV, EZE + ATV and ETD significantly decreased the aortic TNF-α concentrations in comparison to the untreated OVX + TPO.

### Effect of selected antihyperlipidemic drugs and estradiol on aortic markers of oxidative stress in ovariectomized rats fed with TPO and TSO

**Fig. 3:**
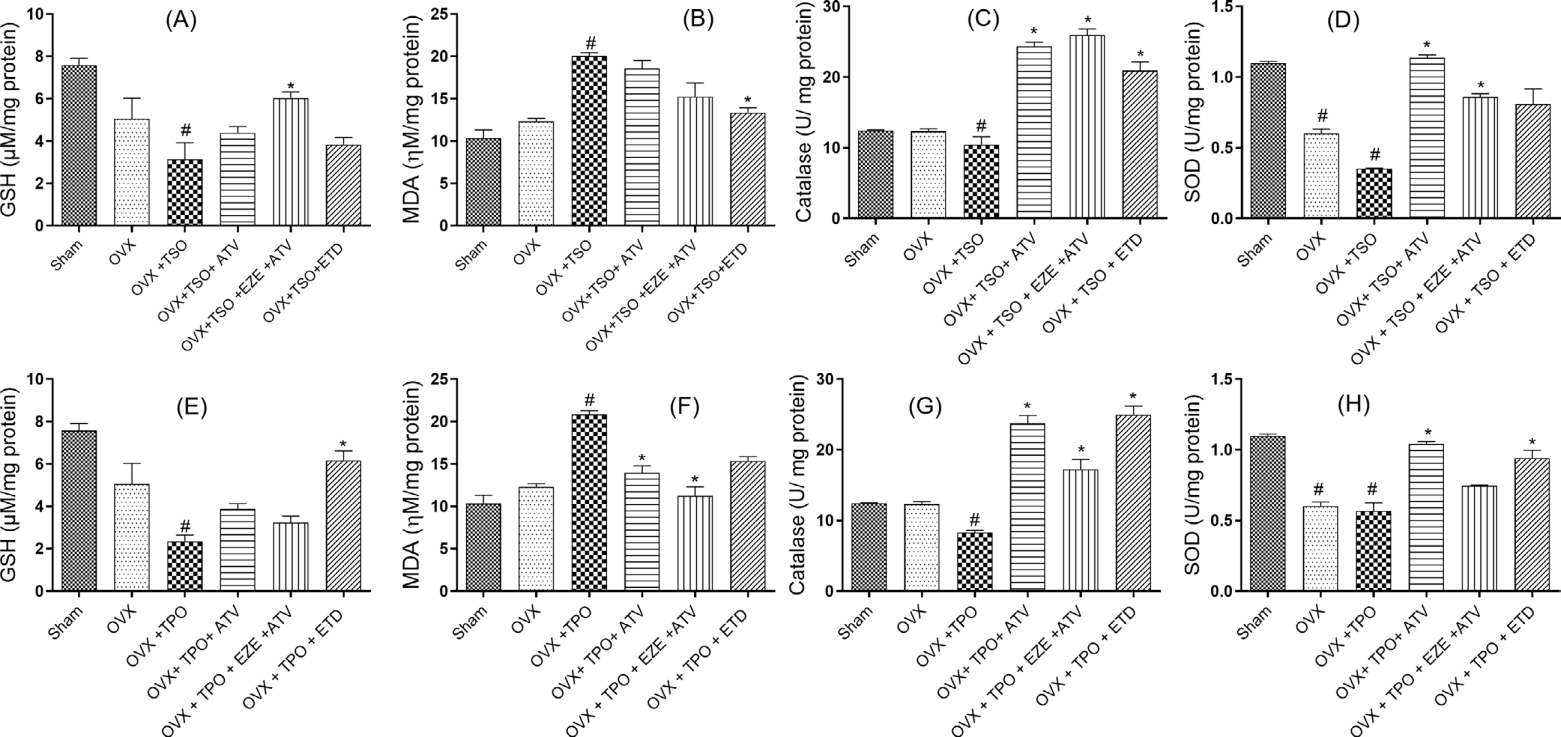
**Effects of estradiol and selected antihyperlipidemic drugs on aortic oxidative stress markers in ovariectomized Wistar rats fed with thermoxidized soya oil (A-D) and thermoxidized palm oil diets (E-H)** Values are expressed as mean ± standard error of mean (n = 3) #*p* < 0.05 compared to Sham accepted as significant *p < 0.05 compared to OVX + TSO/ OVX + TPO **p < 0.01 compared to OVX + TSO/ OVX + TPO ***p < 0.001 compared to OVX + TSO/ OVX + TPO *OVX – ovariectomized; TSO – thermoxidized soya oil; TPO – thermoxidized palm oil; ATV – atorvastatin; EZE – ezetimibe; ETD – estradiol; GSH – glutathione; MDA – Malondialdehyde; CAT – catalase; SOD – superoxide dismutase*

Repeated oral treatments of ovariectomized rats with TSO significantly (*p* < 0.05) reduced GSH levels when compared to Group I values (Fig. 3). Among the treatment groups, ATV + EZE treated rats showed a significant increase in their GSH levels in comparison with the untreated ovariectomized rats treated with TSO. Similarly, the MDA levels were increased (*p* < 0.05) by two folds in the ovariectomized rats treated with TSO when compared with sham. ETD decreased (*p* < 0.05) the MDA levels in comparison with OVX + TSO. CAT levels were significantly decreased in the OVX + TSO group compared to sham. Treatment with ATV, EZE + ATV and ETD all increased (P < 0.05) the CAT levels when compared to rats in the untreated OVX + TSO group. In the EZE + ATV treated rats, there was a 150% increase in CAT levels. SOD levels decreased significantly (*p* < 0.05) in both OVX and OVX + TSO rats compared with sham rats, however, SOD was decreased more in OVX + TPO group compared to the OVX group, although this difference was not significant (*p* > 0.05). All the treatment groups showed significantly elevated SOD levels when compared with the untreated OVX + TSO. ATV treated rats showed a profound (*p* < 0.05) increase in the SOD levels of more than three folds when compared with OVX + TSO. GSH concentration in the untreated OVX + TPO was significantly reduced compared to sham. ETD treatment increased the GSH concentration significantly (*p* < 0.05) when compared with the untreated OVX + TPO group. MDA concentration increased significantly in the OVX + TPO group by 100% when compared with Group I values. However, all the treatment groups ATV, EZE + ATV and ETD decreased MDA concentration significantly (*p* < 0.05) when compared to the untreated TSO-fed ovariectomized rats. CAT concentration decreased significantly (*p* < 0.05) in the untreated TSO-fed ovariectomized rats when compared with Group I values. Conversely, ATV, EZE + ATV and ETD treatment increased CAT levels significantly when compared with the untreated TPO-fed ovariectomized rats. In ETD treated rats, there was a threefold increase in CAT levels. SOD levels decreased significantly (*p* < 0.05) in the untreated OVX as well as OVX + TPO rats when compared to sham. However, SOD was further decreased in OVX + TPO group compared to the OVX group albeit insignificantly (*p* > 0.05). Treatment groups ATV and ETD raised the SOD levels significantly (*p* < 0.05) when compared with the untreated TPO-fed ovariectomized rats.

### Effect of selected antihyperlipidemic drugs and estradiol on the histology of ascending and thoracic aorta of ovariectomized rats fed with TPO and TSO

**Fig. 4:**
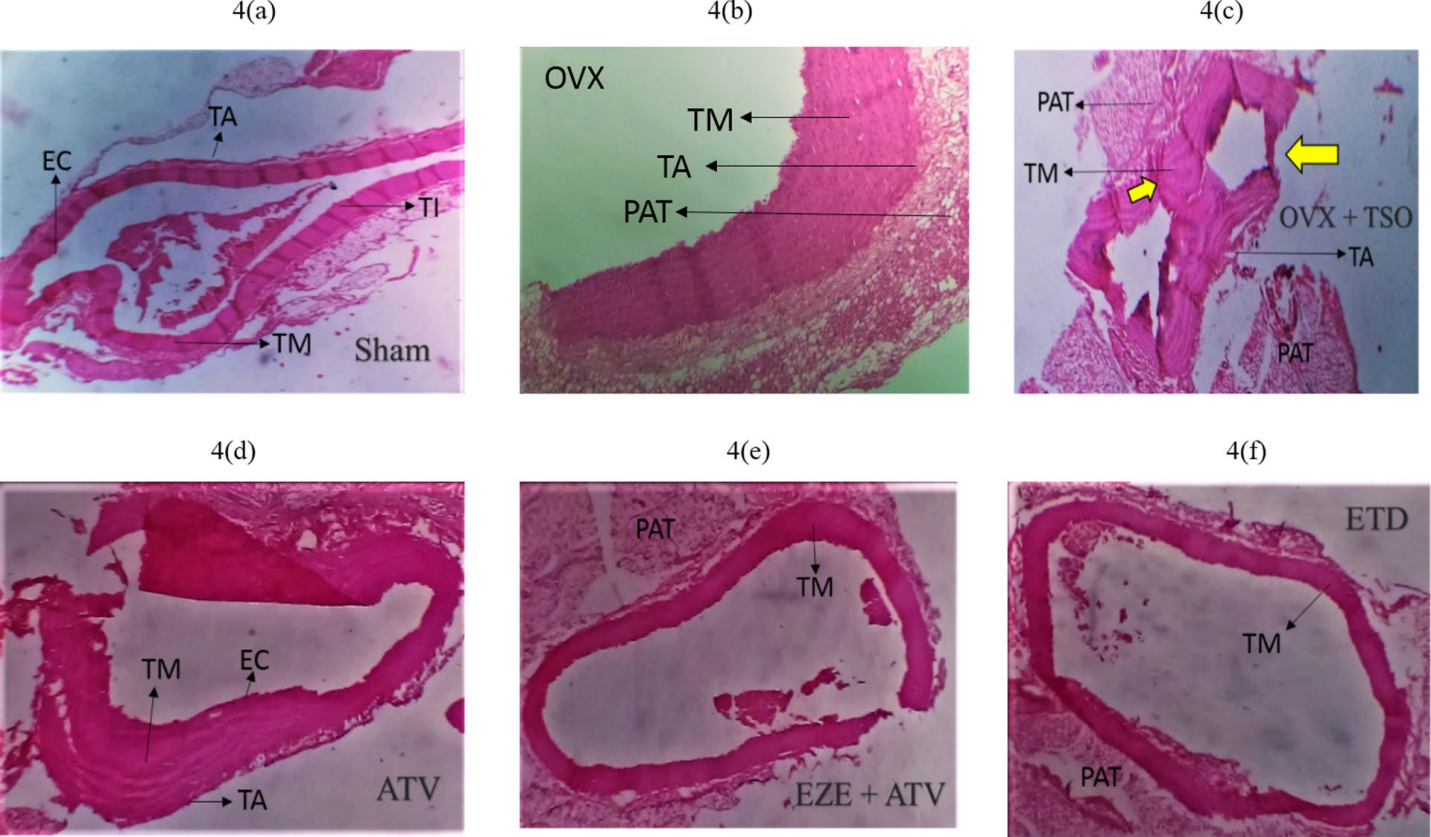
**Photomicrographs of the effects of estradiol and selected antihyperlipidemic drugs on the ascending aorta of ovariectomized Wistar rats fed with thermoxidized soya oil diet. (4a) Sham (4b) OVX (4c) OVX + TSO (4d) ATV (4e) EZE + ATV (4f) ETD** *Note* the thickening of the tunica media (TM) indicated by the small yellow arrow and aortic recanalization indicated by the big yellow arrow in OVX + TSO (c). *Note* the reduced amount of red blood cells in the luminal aspect of the aortic wall and the preservation of the elastic laminae of the tunica media indicated by TM in ATV, EZE + ATV and ETD treatment groups. H&E staining of the rat aorta (x100 magnification) *OVX – ovariectomy; TSO – thermoxidised soya oil; EC – endothelial cells; TM – tunica media; TI – tunica intima; TA – tunica adventitial; PAT – peri-adventitial fat; ATV – atorvastatin; EZE – ezetimibe; ETD – estradiol*

**Fig. 5:**
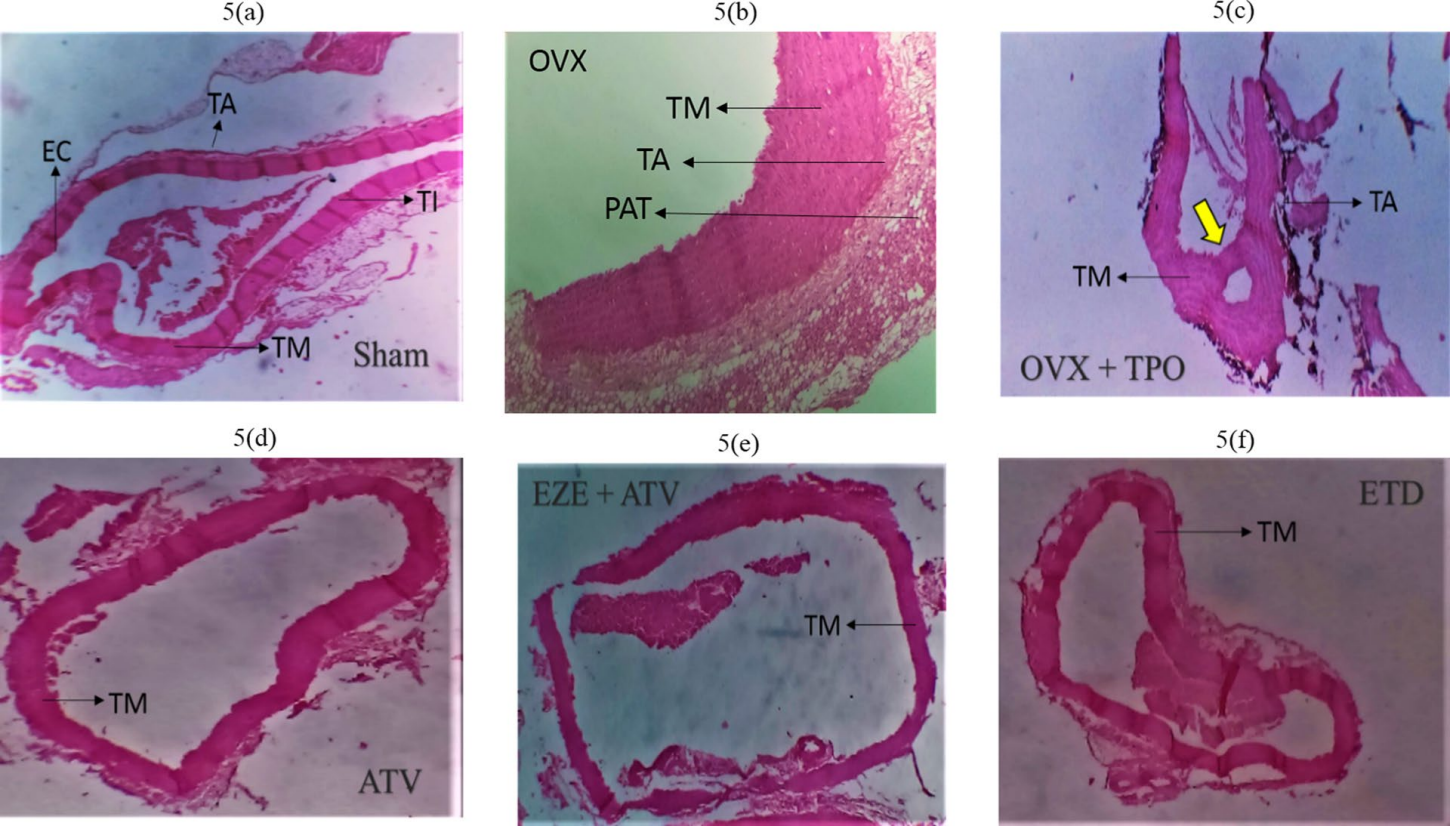
**Photomicrographs of the effects of estradiol and selected antihyperlipidemic drugs on the ascending aorta of ovariectomized Wistar rats fed with thermoxidized palm oil diet. (5a) Sham (5b) OVX (5c) OVX + TPO (5d) ATV (5e) EZE + ATV (5f) ETD** *Note* the thickening of the tunica media indicated by TM and aortic recanalization indicated by the yellow arrow in OVX + TPO. *Note* the reduced amount of red blood cells in the luminal aspect of the aortic wall and the preservation of the elastic laminae of the tunica media indicated by TM in ATV, EZE + ATV and ETD treatment groups. H&E staining of the rat aorta (x100 magnification) *OVX – ovariectomy; TPO – thermoxidised palm oil; EC – endothelial cells; TM – tunica media; TI – tunica intima; TA – tunica adventitial; PAT – peri-adventitial fat; ATV – atorvastatin; EZE – ezetimibe; ETD – estradiol*

Histopathological examination of the ascending aorta of untreated TSO-fed ovariectomized rats showed a general disruption in the histoarchitecture of the aorta coupled with a large amount of red blood cell deposits in the luminal part of the arterial wall (Fig. 4b). These disruptions included associated tunica media (TM) hypertrophy and increased peri-adventitia fat (PAT) (Fig. 4b and c) when compared to normal histoarchitecture in the Group I rats (Fig. 4a). ATV, EZE + ATV and ETD treatment groups all showed essentially normal histoarchitecture of the aorta (Fig. 4d–f). Similarly, the aortas of the untreated TPO-fed ovariectomized rats showed deposition of red blood cells in the luminal region of the arterial wall as well as hypertrophy of the peri-adventitia adipose tissue (Fig. 5c) when compared to normal histoarchitecture in the Group I rats (Fig. 5a). The Aorta of ATV, EZE + ATV and ETD treated rats fed with TPO diet all showed essentially normal histology (Fig. 5d–f).

**Fig. 6:**
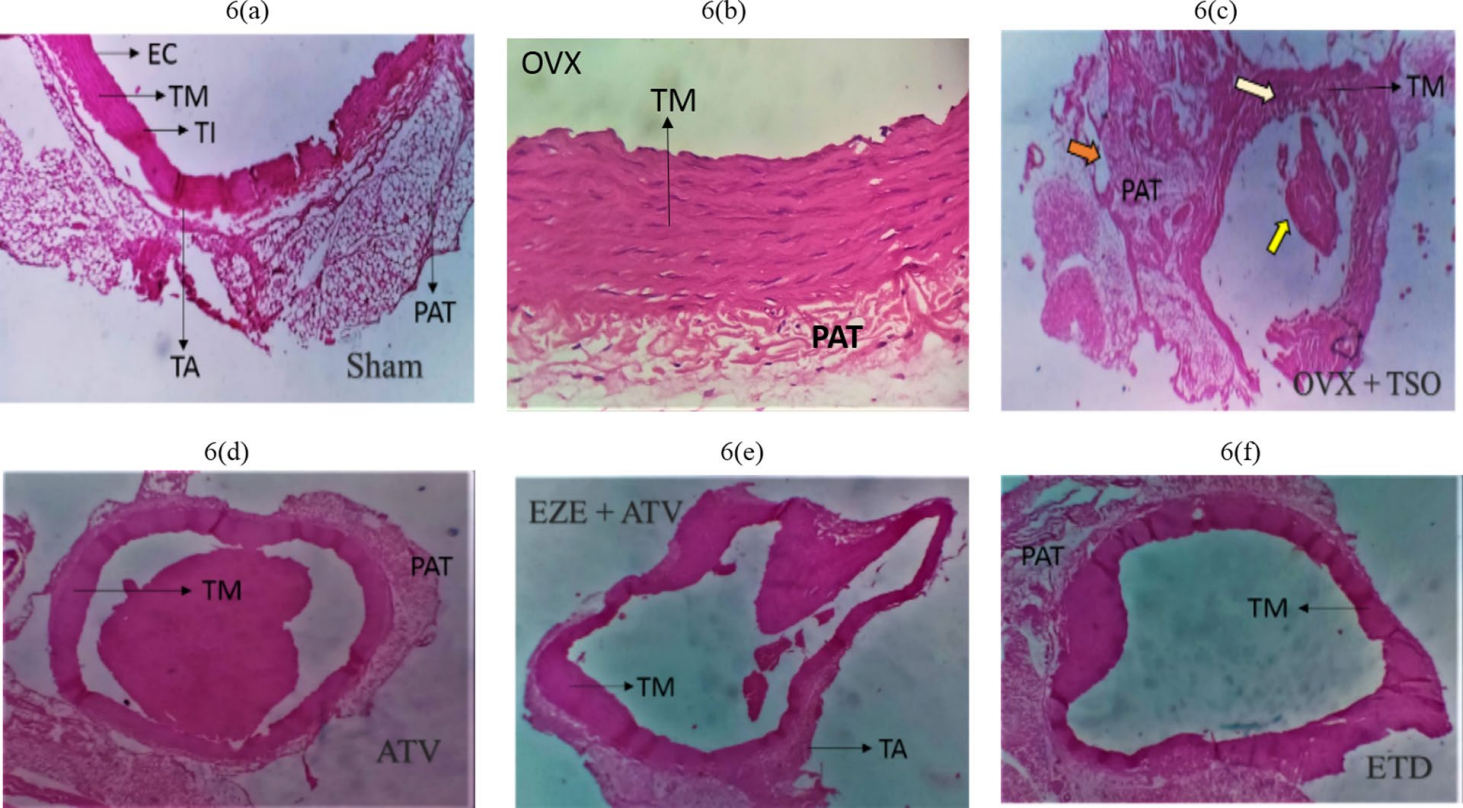
**Photomicrographs of the effects of estradiol and selected antihyperlipidemic drugs on the thoracic aorta of ovariectomized Wistar rats fed with thermoxidized soya oil diet. (6a) Sham (6b) OVX (6c) OVX + TSO (6d) ATV (6e) EZE + ATV (6f) ETD** *Note* the accumulation of large amount of red blood cells indicated by the yellow arrow; thickness of tunica media and complete disruption of the elastic laminae indicated by the white arrow and increased deposition of peri-adventitial fat (PAT) indicated by the red arrow in (c). *Note* the preservation of the elastic laminae of the tunica media, indicated by TM in (d-f). H&E staining of the rat aorta (x100 magnification) *OVX – ovariectomy; TSO – thermoxidized soya oil; EC – endothelial cells; TM – tunica media; TI – tunica intima; TA – tunica adventitia; PAT – peri-adventitial fat; ATV – atorvastatin; EZE – ezetimibe; ETD – estradiol*

**Fig. 7:**
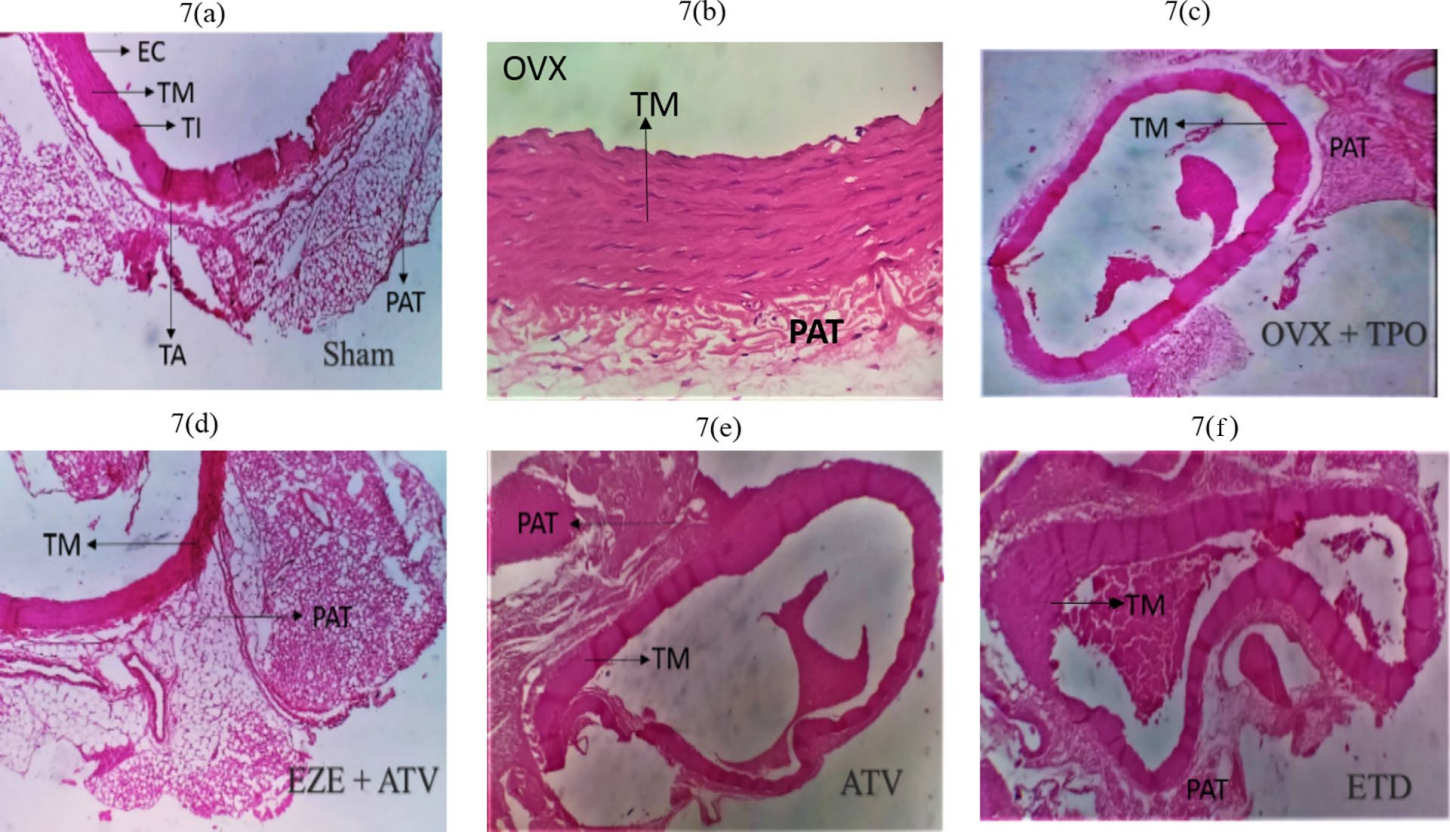
**Photomicrographs of the effects of estradiol and selected antihyperlipidemic drugs on the thoracic aorta of ovariectomized Wistar rats fed with thermoxidized palm oil diet. (7a) Sham (7b) OVX (7c) OVX + TPO (7d) EZE+ATV (7e) ATV (7f) ETD** *Note* hypertrophy of the peri-adventitial adipose tissue indicated by the black arrow in (OVX + TPO). *Note* the preservation of the elastic laminae of the tunica media, indicated by TM in all treatment groups. H&E staining of the rat aorta (x100 magnification) *OVX – ovariectomy; TPO – thermoxidized palm oil; EC – endothelial cells; TM – tunica media; TI – tunica intima; TA – tunica adventitia; PAT – peri-adventitial fat; ATV – atorvastatin; EZE – ezetimibe; ETD – estradiol*

The thoracic aorta of OVX + TSO rats (Fig. 6b) revealed recanalization with tunica hypertrophy. Sham aorta revealed normal histoarchitecture (Fig. 6a). The endothelium, tunica intima, tunica media and tunica adventitia were observed to be normal (Fig. 6a). ATV, EZE + ATV and ETD treatment all showed essentially normal histology of the aorta (Fig. 6d–f). Thoracic Aorta of OVX + TPO (Fig. 7c) rats showed recanalization with marked endothelial disruption, tunica media hypertrophy, tunica adventitia disruption, as well as increased peri-adventitia fat when compared to normal histoarchitecture in the Group I rats (Fig. 7a). Thoracic aortas of ATV, EZE + ATV and ETD-treated rats fed showed essentially normal histoarchitecture (Fig. 7d–f).

## Discussion

Selected antihyperlipidemic drugs and estrogen prevented hyperlipidemia, aortic tissue inflammation and oxidative stress, in ovariectomized rats fed with thermoxidized oil diets. Deep fat frying with palm and Soybean oil initiated chemical reactions that resulted in the generation of oxidized products while also reducing the antioxidant contents of the oil [11]. These reactive products can activate inflammatory processes that culminate in diminished vascular integrity and cardiovascular health. These effects, coupled with estrogen withdrawal in postmenopausal women significantly increase the risk for CVD [31–34].

Chronic consumption of thermoxidized palm/soya oil in postmenopausal rats has been reported to cause atherogenic dyslipidemia through the elevation of total cholesterol, triglycerides, LDL-c, and decreased the HDL-c [35–37]. In this study, palm and soya oils supplemented diet fed to ovariectomized rats significantly increased atherogenic dyslipidemia. Treatment with antihyperlipidemic drugs and estradiol reversed indices of atherogenic dyslipidemia in ovariectomized rats fed with palm and soya oil-supplemented diets. The association between hyperlipidemia and atherosclerosis including the ensuing incidence of cardio-cerebrovascular diseases is well established in the literature [35, 38, 39]. ETD, a major component of the therapeutic regimen is widely used in the treatment of postmenopausal hormonal deficiency syndrome [40]. The effect of ETD on serum and lipoprotein lipids is characterized by an increase in the lipid constituents of high-density lipoproteins (HDL) and, usually, a decrease in low-density lipoproteins (LDL). LDL-c reduction by these treatments shows that they offer significant protection against atherosclerosis [40–42]. According to the European Society of Cardiology (ESC) and European atherosclerosis society (EAS) guidelines for the management of dyslipidemia, 1 mmol/L reduction in LDL cholesterol level corresponds to a one-fifth reduction in the risk of atherosclerotic cardiovascular disease (ASCVD) [43]. When evaluated for other lipid parameters, ATV, EZE + ATV and ETD treatments decreased the TG levels, while ATV increased the HDL-c levels. The mechanisms underlying the lipid-controlling effect of these antihyperlipidemic drugs have been widely described in the literature [43]. ATV inhibits HMG-CoA reductase while EZE inhibits dietary and biliary cholesterol uptake in the intestine [43]. In OVX + TPO rats, ATV decreased the TC levels significantly while ATV and EZE + ATV significantly decreased TG and VLDL-c levels. Estradiol reduces LDL cholesterol levels and increases HDL cholesterol levels in postmenopausal women with normal or elevated baseline lipid levels [44, 45]. The reduction in LDL cholesterol levels is probably a result of the accelerated conversion of hepatic cholesterol to bile acids and increased expression of LDL receptors on cell surfaces, resulting in augmented clearance of LDL from the plasma [44, 45].

A search of the literature shows evidence of increased cardiovascular risk and incidence of adverse events associated with a decrease in the HDL/LDL cholesterol ratio [46, 47]. TSO and TPO decreased the HDL/LDL ratio albeit insignificantly while also elevating the AI and CRI significantly (*p* < 0.05). The atherogenic index is a strong marker for assessing the risk of atherosclerosis and coronary heart disease in vessels. As AI increases, the risk of atherogenesis increases [48, 49]. Estradiol and the antihyperlipidemic drugs employed in this study significantly decreased the AI and CRI. ATV, EZE + ATV and ETD administration significantly decreased the AI and/or CRI. This decrease in the atherogenic indices demonstrates the beneficial effect of early administration of antihyperlipidemic drugs and estrogen in the reduction or prevention of atherogenesis. In rats fed with TSO diet, ATV treatment significantly increased the HDL/LDL cholesterol ratio by approximately three folds. An increase in the HDL/LDL cholesterol ratio is an indicator of low circulating LDL in the plasma, which is beneficial in the prevention of atherosclerotic vascular disease [43, 50]. In rats fed with TPO diet, ATV administration significantly decreased the AI and CRI, while ETD and EZE + ATV decreased AI significantly.

Aortic nitric oxide was examined as an indicator of vascular integrity in this study. In both TPO and TSO-fed ovariectomized rats, aortic nitric oxide levels were reduced. This effect was reversed in antihyperlipidemic, and estrogen treated rats. Diminished NO is associated with endothelial dysfunction and predisposes the vessel to atherosclerosis [51, 52]. The elevation of NO through the upregulation of endothelial nitric oxide synthase (eNOS) plays an important role in preventing or reversing endothelial dysfunction associated with hypertension, atherosclerosis and other cardiovascular diseases [53, 54]. Estrogen and the combination of ATV and EZE reportedly increased aortic nitric oxide levels, possibly via the up-regulation of eNOS as earlier demonstrated for the individual drugs [55, 56].

TNF-α-activated signaling contributes to vascular dysfunction, development and progression of atherosclerosis [9]. The pro-atherogenic effects of TNF-α on the endothelium include reactive oxygen species (ROS) production while increased TNF-α levels impair endothelium-dependent vasodilation in a NO-dependent manner [9]. Evaluation of TNF-α levels in the tissues of the thoracic aorta revealed a significant increase in TNF-α levels of TSO and TPO fed rats. This indicates that chronic consumption of thermoxidised oils results in diminished endothelial and, by extension, vascular integrity. Prolonged increase in aortic TNF-α concentration initiates expression of pro-inflammatory, pro-atherogenic proteins that contribute to increased recruitment and transmigration of circulating leukocytes into the vascular wall. These processes are deleterious to vascular health and contribute to atherogenesis [9]. In both TSO and TPO fed rats, the antihyperlipidemic drugs and estradiol decreased TNF-α levels significantly. Results from this study suggest that inhibition of TNF-α signaling is a possible mechanism for the prevention of endothelial dysfunction and atherosclerosis progression by these drugs.

The role of oxidative stress in aortic damage progression has been widely described in the literature [57, 58]. It has been reported to involve the generation of ROS and reactive nitrogen species (RNS) through different pathways such as mitochondrial xanthine oxidase, NADPH oxidase (Nox) or endothelial nitric oxide synthase (eNOS). The thermal oxidation of edible oils decreases the antioxidant content and increases lipid peroxidation and generation of ROS [35]. Oxidative stress occurs when the cellular antioxidant system is overwhelmed by pro-oxidant molecules. Vascular oxidative stress is one of the leading causes of cardiovascular diseases, including atherosclerosis. Elevated oxidative stress results in vasoconstriction, vascular remodeling, inflammation, and fibrosis [59, 60]. MDA is a product of lipid peroxidation and a marker of oxidative stress while GSH, catalase and SOD are important components of the antioxidant system. Lipid peroxidation is associated with the endothelial dysfunction involved in atherosclerosis and plays an important role in the mechanism of immune response to vascular injury [61]. Results from this study suggest that the chronic consumption of TSO and TPO diet elevates oxidative stress through the generation of ROS and the deficiency of the antioxidant system, indicated by the significant increase in aortic MDA levels, as well as the significant decrease in the GSH, SOD and catalase in the untreated OVX + TSO and OVX + TPO rats. ETD appears to reduce the levels of lipid peroxidation and generation of ROS in both TSO and TPO fed rats, as shown by a significant decrease in MDA levels. On the other hand, Catalase and SOD levels were significantly increased by these treatments as well as the ATV and EZE + ATV treatments. Similarly, aortic GSH level was significantly increased by EZE + ATV treatment. Estrogen modulates genes involved in the regulation of vascular tone and response to vascular injury. Estrogen binding to the vascular receptor stimulates the expression of genes for enzymes that participate in vasodilation such as Nitric oxide synthase and prostacyclin synthase [62]. This result indicates a replenishment of the antioxidant system by the respective treatments. Replenishing the antioxidant system helps to combat oxidative stress, thus reducing or preventing tissue damage. An increase in the expression of SOD has been reported to offer vascular protection including the inhibition of vascular hypertrophy [63].

In rats fed with TPO diet, ATV, EZE + ATV and ETD treatments offered protection against oxidative stress, as shown by the significant decrease in aortic tissue MDA levels while ATV, EZE + ATV and ETD treatments replenished the antioxidant system as shown by the significant increases in one or more of aortic tissue concentrations of GSH, CAT, and SOD.

Histological evaluation of the ascending aorta of the untreated OVX + TSO rats showed evidence of atherosclerotic changes. Accumulation of numerous blood cells were observed in the luminal aspect of the aortic wall, indicating inflammation in the aorta. The process of vascular inflammation includes stasis of blood flow and vascular congestion in the lumen of the aorta, leukocyte margination and adhesion along the endothelium, diapedesis and phagocytosis [44, 64–66].

Further examination revealed hypertrophy of the tunica media and increased peri-adventitial fat in the aorta of the untreated OVX + TSO. Hypertrophy is a form of cellular adaptation that involves an increase in cell size in response to an external stimulus such as prolonged inflammation and mechanical stress. Aortic medial hypertrophy impacts the elasticity of the vessel and can result in blood pressure elevation and CVD [6]. Although the peri-adventitial adipose tissue has been found to offer protection to the vessels, persistent increases in overweight/obese subjects result in adipocyte dysfunction. Dysfunctional adipocytes are implicated in the immune response and vascular smooth muscle cell proliferation involved in the progression of atherosclerosis [67]. Examination of the thoracic aorta of OVX + TSO rats revealed recanalization of the aorta along with arterial medial hypertrophy and increased peri-adventitia fat. This pattern of atherosclerotic changes was also observed in the ascending and thoracic aorta of rats fed with TPO, as shown by the accumulation of red blood cells in the intimal part of the ascending aorta coupled, with increased peri-adventitia fat, and recanalization of the descending aorta, with tunica media hypertrophy. Examination of the aorta of rats in all the treated groups of both TSO and TPO rats revealed restoration to normal histoarchitecture.

## Conclusions

Taken together, chronic consumption of thermoxidized palm/soya oil is a risk factor for the development and progression of atherosclerosis in post-menopausal Wistar rats. This is due to its role in elevating atherogenic lipid profile, oxidative stress and vascular damage. Early administration of estrogen and antihyperlipidemic drugs offers protection to the vessels and may prevent the development and progression of atherosclerosis.

## Data Availability

All data generated or analyzed during this study are included in this published article.

## List of Abbreviations

AHD: antihyperlipidemic drugs
ETD: estradiol/estradiol valerate/estrogen
TPO: thermoxidized palm oil
TSO: thermoxidized soya oil
OVX: ovariectomized
ATV: Atorvastatin
BP: Blood pressure
SBP: Systolic blood pressure
DBP: Diastolic blood pressure
MAP: Mean arterial pressure
QTc: QT corrected
HDL-c: high-density lipoprotein
LDL-c: low density lipoprotein
T-CHOL: Total cholesterol
VLDL: very low density lipoprotein
TG: triglycerides
AI: atherogenic index
CRI: Coronary risk index
ECG: electrocardiogram
PUFA: polyunsaturated fatty acids
SF: Saturated fats
CVD: cardiovascular disease
ACS: acute coronary syndromes
MDA: Malondialdehyde
TBARS: Thiobarbituric reacting substances
GSH: Reduced glutathione
CAT: Catalase
SOD: superoxide dismutase
SEM: standard error of mean
ANOVA: analysis of variance
Ox-LDL: oxidized low-density lipoprotein
ROS: reactive oxygen species
NO: nitric oxide
